# Supplementary figures and images for: Characteristics of aldosterone-producing adenomas in patients without plasma renin activity suppression
Source: PLoS One. 2022 Apr 28;17(4):e0267732. doi: 10.1371/journal.pone.0267732 (PMC9049528; doi:10.1371/journal.pone.0267732)

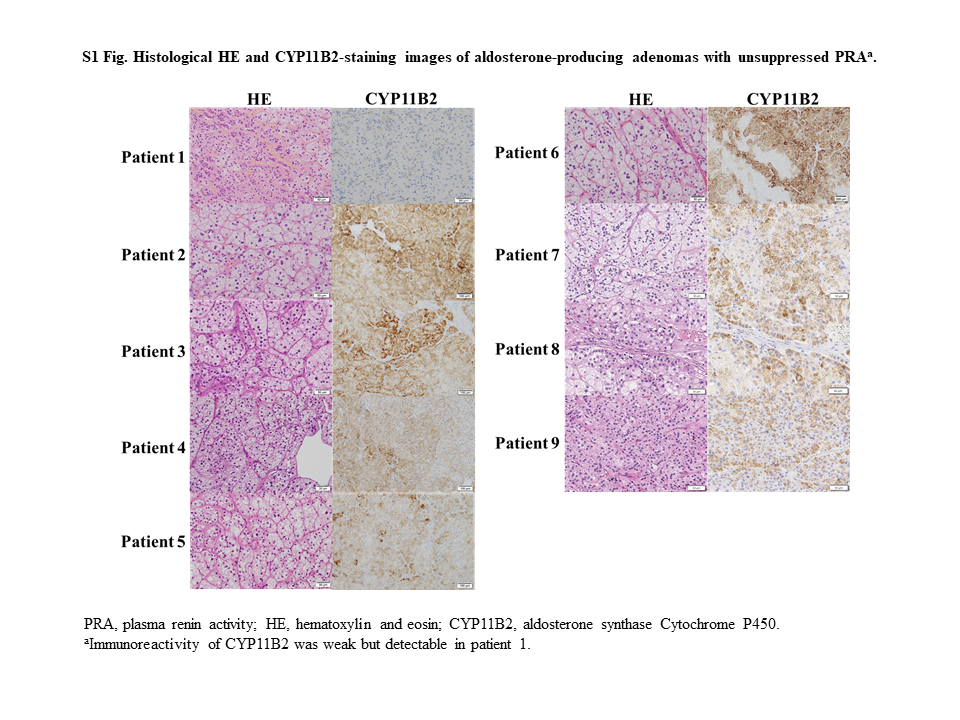

Supplement: S1 Fig — (TIF) [file pone.0267732.s001.tif]
