# Supplementary material for: Characteristics of aldosterone-producing adenomas in patients without plasma renin activity suppression
Source: PLoS One. 2022 Apr 28;17(4):e0267732. doi: 10.1371/journal.pone.0267732 (PMC9049528; doi:10.1371/journal.pone.0267732)
Supplement: S1 Table — (DOCX) [file pone.0267732.s002.docx]

**S1 Table. Baseline clinical characteristics of each patient in the unsuppressed PRA group.**

|  | Patient 1 | Patient 2 | Patient 3 | Patient 4 | Patient 5 | Patient 6 | Patient 7 | Patient 8 | Patient 9 |
| --- | --- | --- | --- | --- | --- | --- | --- | --- | --- |
| Sex | Male | Female | Male | Male | Female | Female | Female | Female | Male |
| Age (years) | 57 | 51 | 55 | 46 | 57 | 56 | 47 | 35 | 46 |
| BMI (kg/m^2^) | 28.7 | 20.7 | 24.9 | 25.6 | 28.0 | 18.4 | 28.7 | 18.9 | 19.6 |
| Systolic blood pressure (mmHg) | 143 | 130 | 178 | 162 | 134 | 129 | 133 | 120 | 116 |
| Diastolic blood pressure (mmHg) | 91 | 90 | 108 | 108 | 89 | 77 | 84 | 79 | 72 |
| Duration of hypertension (years) | 17 | 21 | 10 | 10 | 27 | 10 | 15 | 6 | 2 |
| History of cardiovascular disease | Stroke | Stroke | (-) | (-) | (-) | (-) | (-) | (-) | (-) |
| Diabetes mellitus^a^ | (+) | (-) | (+) | (+) | (+) | (-) | (+) | (-) | (-) |
| Dyslipidemia | (+) | (-) | (-) | (+) | (-) | (-) | (+) | (-) | (-) |
| Number of antihypertensive drugs | 2 | 1 | 2 | 2 | 1 | 1 | 1 | 2 | 2 |
| Serum potassium (mEq/L) | 3.6 | 3 | 3.6 | 3.2 | 3.7 | 2.7 | 2.2 | 3.2 | 3.4 |
| Oral potassium supplements, n (%) | (+) | (+) | (-) | (-) | (-) | (+) | (+) | (+) | (+) |
| eGFR (mL/min/1.73 m^2^) | 46.8 | 91.0 | 85.4 | 55.5 | 39.7 | 96.4 | 61.5 | 92.0 | 80.3 |
| Urine albumin (mg/g・Cr) | 70.83 | 14.9 | 6.8 | 16.7 | 14.7 | 18.1 | 40.0 | 22.1 | 10.5 |
| baPWV (m/sec) | 1826.5 | 1276.5 | 1660 | 1516 | 1232.5 | NA | 1481 | 1586.5 | 1261 |
| ^c^Apnea-hypo index | 44.0 | 25.8 | 13.4 | NA | NA | NA | 26.4 | 3.0 | NA |
| Adrenal tumor in CT scan | 26 mm lt | not detectable | 11 mm lt | 8 mm lt, 9mm lt | 10 mm lt | 20 mm rt | 28 mm rt | 14 mm rt | 8mm rt, 14 mm lt |
| Adrenalectomy procedure | lt total | rt total | rt partial | lt total | lt partial | rt total | rt partial | rt partial | rt partial |
| Clinical outcome 1 year after surgery^b^ | absent | partial | partial | partial | partial | complete | partial | partial | partial |

PRA, plasma renin activity; BMI, body mass index; eGFR, estimated glomerular filtration rate; baPWV, brachial-ankle pulse wave velocity; CT, computed tomography; lt, left; rt, right; NA, not applicable.

^a^Diabetes mellitus included borderline diabetes mellitus.

^b^Clinical outcomes were evaluated according to the Primary Aldosteronism Surgical Outcomes criteria [23].

^C^ We investigated the degree of sleep apnea using a 2-channel (airflow and SpO2) portable sleep apnea monitor (LS-120/120S, Fukuda Denshi Co, Ltd, Tokyo, Japan).)
