# Supplementary material for: Characteristics of aldosterone-producing adenomas in patients without plasma renin activity suppression
Source: PLoS One. 2022 Apr 28;17(4):e0267732. doi: 10.1371/journal.pone.0267732 (PMC9049528; doi:10.1371/journal.pone.0267732)
Supplement: S2 Table — (DOCX) [file pone.0267732.s003.docx]

**S2 Table. Endocrine parameters of each patient in the unsuppressed PRA group.**

|  | Patient 1 | Patient 2 | Patient 3 | Patient 4 | Patient 5 | Patient 6 | Patient 7 | Patient 8 | Patient 9 |
| --- | --- | --- | --- | --- | --- | --- | --- | --- | --- |
| PRA (ng/mL/hr) | 2.5 | 1.6 | 2.5 | 1.7 | 2.5 | 1.8 | 1.4 | 2.9 | 1.5 |
| PAC at 8:00 (ng/dL) | 24.0 | 18.9 | 12.8 | 34.3 | 18.1 | 83.3 | 160.5 | 121.7 | 46.6 |
| ARR | 9.6 | 6.3 | 5.1 | 20.2 | 7.2 | 46.2 | 114.6 | 42.0 | 31.0 |
| Cortisol at 8:00 (μg/dL) | 13.2 | 10.5 | 7.1 | 12.6 | 7.6 | 9.3 | 12.8 | 20.4 | 13.8 |
| Cortisol at 23:00 (μg/dL) | 3.4 | 2.3 | 1.7 | 4.0 | 1.8 | 5.5 | 3.1 | 4.7 | 4.2 |
| ACTH at 8:00 (pg/mL) | 10.8 | 15.3 | 36.4 | 13.6 | 14.0 | 14.3 | 8.2 | 59.2 | 17.1 |
| ACTH at 23:00 (pg/mL) | 9.5 | 5.1 | 10 | 8.7 | 9 | 4.5 | 3.8 | 8.7 | 6.2 |
| Urinary aldosterone (μg/day) | 22.4 | 14.2 | 18.5 | 16.8 | 14.4 | 6.1 | 35.3 | 67.8 | 21.4 |
| Urinary cortisol (μg/day) | 30.2 | 68.9 | 74.1 | 45.5 | 39.3 | 25.0 | 52.2 | 69.1 | 48.3 |
| PAC (240 min after saline loading) | 20.8 | 14.3 | NA | 24.7 | 18.5 | 47.7 | 255.0 | 77.4 | 49.1 |
| PRA (120 min after furosemide loading) | NA | NA | 4.4 | 3.4 | 3.2 | NA | NA | 6.7 | 2.9 |
| ARR (90 min after captopril loading)^a^ | 5.3 | 12.9 | 6.0 | 15.3 | 5.6 | 46.5 | 88.4 | 47.0 | 49.5 |
| Max PAC/cortisol ratio after ACTH stimulation | 2.73 | 2.64 | 1.42 | 3.09 | 2.2 | 9.0 | 14.3 | 4.8 | 2.0 |
| Cortisol level after 1 mg DST (μg/dL) | 2.3 | 1.7 | 1.1 | 1.5 | 2.0 | 2.6 | 7.5 | 2.2 | 2.2 |

PRA, plasma renin activity; PAC, plasma aldosterone concentration; ARR, aldosterone/renin ratio; ACTH, adrenocorticotropic hormone; DST, dexamethasone suppression test; SCS, subclinical Cushing’s syndrome; NA, not applicable.

Conversion to SI units: PAC, ng/dL × 27.7 for pmol/L; Cortisol, μg/dL × 27.6 for nmol/L; ACTH, pg/mL × 0.220 for pmol; Urinary aldosterone, μg/day × 2.77 for nmol/day; Urinary cortisol, μg/day × 2.76 for nmol/day.

^a^ARR is calculated as PAC (ng/dL) divided by PRA (ng/mL/hr).

Not all of them fulfilled the criteria of diagnosis in PA and diagnosed with comprehensive assessment followings. The results of the furosemide loading test and the captopril loading test were negative except patient 8 probably due to unsuppressed PRA and their diagnosis was done comprehensively deviated from guidelines. Finally, sAVS lead to definite diagnosis of hyperaldosteronism and reviewed from pathological findings in surgical specimen of aldosterone producing adenoma [8]. Especially, patient 3 underwent sAVS and surgical procedure judged in a comprehensive manner following; uncontrollable hypertension (178/108 mmHg with 2 antihypertensive drugs), relatively hypokalemia (K 3.6 mEq/L), positive data in rapid-ACTH test and complication of diabetes which means poor prognosis.
