# Supplementary material for: Characteristics of aldosterone-producing adenomas in patients without plasma renin activity suppression
Source: PLoS One. 2022 Apr 28;17(4):e0267732. doi: 10.1371/journal.pone.0267732 (PMC9049528; doi:10.1371/journal.pone.0267732)
Supplement: S3 Table — (DOCX) [file pone.0267732.s004.docx]

**S3 Table. Results of segment-selective adrenocorticotropic hormone-loading adrenal venous sampling in the unsuppressed PRA group.**

|  | Right Adrenal Vein | | | |  | Left Adrenal Vein | | | | LI | CR | Adrenal tumor in CT scan | Surgical side | Histological evaluation |
| --- | --- | --- | --- | --- | --- | --- | --- | --- | --- | --- | --- | --- | --- | --- |
|  | Central Venous | Superior Branch | Lateral Branch | Inferior Branch |  | Central Venous | Superior Branch | Lateral Branch | Inferior Branch |  |  |  |  |  |
| Patient 1 | **2760** | **3230** | **3380** | **4740** |  | 864 | **2380** | **11300** | NA | 6.5 | 2.1 | 26 mm lt | lt | APA + MAPN |
|  | 478 | 492 | 525 | 596 |  | 970 | 1090 | 401 | NA |  |  |  |  |  |
| Patient 2 | 10100 | **9160** | **25200** | **1500** |  | 756 | 711 | 438 | 270 | 9.1 | 0.6 | not detectable | rt | APA |
|  | 688 | 557 | 632 | 629 |  | 469 | 484 | 499 | 566 |  |  |  |  |  |
| Patient 3 | 1100 | 649 | 759 | **1610** |  | 734 | 852 | 746 | NA | 1.9 | 0.4 | 11 mm lt | rt | APA |
|  | 115 | 152 | 160 | 114 |  | 143 | 157 | 138 | NA |  |  |  |  |  |
| Patient 4 | **1510** | NA | 904 | 831 |  | **4020** | 1140 | NA | **2270** | 3.1 | 0.6 | 8 mm lt, 9mm lt | lt | APA |
|  | 860 | NA | 789 | 751 |  | 746 | 1020 | NA | 974 |  |  |  |  |  |
| Patient 5 | **1760** | 1200 | **4200** | NA |  | **3020** | **3840** | **2030** | NA | 1.3 | 1.3 | 10 mm lt | lt | APA |
|  | 615 | 698 | 1000 | NA |  | 785 | 737 | 829 | NA |  |  |  |  |  |
| Patient 6 | **4010** | **5870** | **21400** | NA |  | 412 | 415 | 383 | NA | 9.7 | 0.1 | 20 mm rt | rt | APA |
|  | 482 | 500 | 540 | NA |  | 480 | 492 | 470 | NA |  |  |  |  |  |
| Patient 7 | **42200** | 823 | **58800** | 1260 |  | NA | NA | NA | NA | NA | NA | 28 mm rt | rt | APA |
|  | 1110 | 1150 | 1050 | 1080 |  | NA | NA | NA | NA |  |  |  |  |  |
| Patient 8 | **1740** | 278 | 341 | **2754** |  | 1340 | 234 | 437 | NA | 1.4 | 0.6 | 14 mm rt | rt | APA |
|  | 456 | 439.9 | 518.8 | 424.7 |  | 476 | 360 | 453 | NA |  |  |  |  |  |
| Patient 9 | **5520** | NA | NA | **16118** |  | **2180** | 1270 | **7920** | NA | 3.7 | 3.7 | 8mm rt, 14 mm lt | rt | APA |
|  | 751 | NA | NA | 848 |  | 1089 | 1335.3 | 1020.3 | NA |  |  |  |  |  |

PRA, plasma renin activity; NA, not available; LI, lateralization index; CR, contralateral ratio; CT, computed tomography; lt, left; rt, right. APA, aldosterone-producing adenoma; LI, lateralization index; MAPN, multiple aldosterone-producing nodules; NA, not applicable.

Conversion to SI units: PAC, ng/dL × 27.7 for pmol/L; Cortisol, μg/dL × 27.6 for nmol/L

Upper values are aldosterone concentration (ng/dL), and lower values are serum-free cortisol (μg/dL). Underlined values meet the diagnostic criteria of aldosterone hypersecretion (≥1,400 ng/dL). Because we judged excess aldosterone by absolute aldosterone value in the adrenal tributary vein, some patients with low lateralization index (LI) (i.e., <2.6) underwent surgery. In patient 1, left adrenalectomy was performed given the prominent high aldosterone level in the left lateral branch, the location of the adrenal tumor, and the severity of PA, although diffuse aldosterone excess was observed in the right adrenal vein. Patient 2 showed higher aldosterone levels on the non-tumor-detectable side. Then we diagnosed unilateral hyperaldosteronism on the right side. Patients 3 and 8 were considered to have unilateral APA despite low LI because the aldosterone in the tributary vein exceeded 1400 ng/dL in the right tributary vein. Patients 5 and 9 showed excess aldosterone on both side of the tributary vein; thus, we assumed that they had bilateral lesions (patient 5; small-sized lesions of aldosterone-producing nodule on the right side and APA on the left side, patient 9; bilateral APA). In patient 7, only samples from the right adrenal vein were available, however considering extremely high aldosterone levels in right central venous and lateral branch consistent with CT-detectable tumor, we performed surgical procedure with informed consent.
