# Supplementary material for: Characteristics of aldosterone-producing adenomas in patients without plasma renin activity suppression
Source: PLoS One. 2022 Apr 28;17(4):e0267732. doi: 10.1371/journal.pone.0267732 (PMC9049528; doi:10.1371/journal.pone.0267732)
Supplement: Attachment — Submitted filename: Response to Reviewers.doc [file pone.0267732.s006.doc]

**Response to Reviewers**

Editor comments:

1. Line 38, taken adrenalectomy, please rephrase

Response: We rephrased as “underwent adrenalectomy” to match the grammar (Page 3, Line 33) and revised the manuscript accordingly.

2. Line 104, you need to name and briefly describe the immunoassays or at least reference them

Response: In order to describe the details of immunoassays of PACs, serum cortisol concentrations, and PRAs, we added the references of previous published relevant studies including standardized guidelines in Japan at Material and Methods Section as follows (Page 5, Lines 104-105) for clarification. We modified the sentence as “radioimmunoassay as previously described [11, 12] and standardly used [9, 10, 13].”

3. Line 146, the number of cases here seems to be wrong. Please correct

Response: We must apologize for this confusing sentence. In our hospital, 969 patients with PA visited our hospital while 2007 and 2020. Within these patients, because of the development and introduction of various up-dated systems, 9 and 86 patients fulfilled the inclusion criteria as unsuppressed and suppressed PRA in this study, respectively.

To clarify the method of the population selection, we modified our sentence as “Among patients with PA visited our hospital while 2007 and 2020, 9 and 86 patients fulfilled the inclusion criteria as unsuppressed and suppressed PRA in this study, respectively.” at Result Section (Page 7, Lines 146-147).

4. Line 228, please briefly describe what you mean by complete and partial success

Response:

Response:

We added the explanation phrase as “This standardized outcome criteria mean remission (complete success), improvement (partial success), and persistence (absent success) of PA after adrenalectomy from the viewpoint of both clinical (mainly about blood pressure and the existence of medications) and biochemical outcomes (mainly about excess of aldosterone and renin suppression), respectively.” at Results Section (Pages 11-12, Lines 251-255) and revised manuscript accordingly.

5. Line 282, suppressed here probably meant to be unsuppressed, please check

Response: We agreed with your comments. In this sentence we meant “The duration of hypertension was relatively longer in some cases in unsuppressed group (suppressed group, 10 [8, 19] vs. unsuppressed group, 8 [2, 16] years, *P*=0.15), but there is no significant difference between two groups.” To clarify our meaning, we fixed our sentence as “However, there were no significant differences in these values between two groups…” at Discussion Section (Page 14, Lines 307-308) and revised manuscript accordingly.

Additionally, we replaced up-dated reference at Discussion Section (Page 13, Lines 289-292) because the previous report was old with language restrictions in Japanese.

6. Line 288, might in the unresected side, please rephrase

Response: We have rephrased the term as suggested at Discussion Section (Page 14, Lines 313).

7. Line 310, suppressed probably meant to be unsuppressed, please check

Response: Thank you for your careful review. We therefore replaced the word with “unsuppressed” (Page 14, Lines 338-339) and revised manuscript accordingly.
