# Supplementary material for: Characteristics of aldosterone-producing adenomas in patients without plasma renin activity suppression
Source: PLoS One. 2022 Apr 28;17(4):e0267732. doi: 10.1371/journal.pone.0267732 (PMC9049528; doi:10.1371/journal.pone.0267732)
Supplement: Attachment — Submitted filename: Review_Comments_to_the_Author-reply.docx [file pone.0267732.s007.docx]

***Point-by-Point Responses to the Comments:***

Dear Editors and Reviewers

Thank you very much for reviewing our manuscript and offering valuable advice.

We have read each of your comments carefully and provided our point-by-point responses below. The manuscript has been revised accordingly, as noted by the page and line numbers.

Additionally, we used Editage (www.editage.com) for English language editing such as grammar and spelling check of the manuscript.

**Reviewer #1:**

*1) In the methods section authors are stating that some patients ‘skipped some confirmatory tests’. What does this mean? Did every patient undergo a confirmatory test? What did authors do if two confirmatory tests showed discordant results? On which guidelines was the diagnosis based (JES? ESE?). Please clarify.*

Reply:

We appreciate your insightful comments. What we intended to convey with this sentence was that we did not conduct saline-loading tests or furosemide loading tests for the patients who would be expected to have an elevated risk of complications from the tests, as previously reported (Eur J Endocrinol. 2019 Feb 1;180(2):R45–R58.)”.

We have described the results of the confirmatory tests in the Table S2. More specifically, one patient was exempted from the saline-loading tests and four patients were exempted from the furosemide loading tests in the unsuppressed PRA group because of severe hypokalemia, a prior history of stroke, or severe hypertension.

We usually diagnose PA based on the JES guidelines in clinical practice for those with one or more positive results in the confirmatory tests with suppressed PRA levels. Meanwhile, in this study, we screened for patients with unsuppressed PRA, which made it difficult for them to meet the diagnostic criteria for PA. Therefore, a diagnosis of PA was not one of the inclusion criteria in this study, as described in our response to the next question.

For the same reason, we did not include the JES guideline for a PA diagnosis as an inclusion criterion in the unsuppressed PRA group, as described in the Methods section (lines 111–125). In particular, we noted that four patients did not meet the criteria for a diagnosis of PA (lines 166–171 and 174–176). Moreover, the text describing the difficulty of diagnosing PA in the patients with unsuppressed PRA has been modified and expanded upon (lines 364–389).

*2) In line 149 authors describe four patients who did not fulfill screening criteria for ARR. Moreover, some of these patients had no pathological screening test either. Did those patients finally belong to the unsuppressed PRA group? Could this have had an impact on the analysis/outcome?*

Reply:

In the unsuppressed PRA group (nine patients), four patients exhibited an ARR < 20 (Table S2), one of whom did not have a positive result in the confirmatory tests.

The study’s inclusion criteria are described in the Methods section (lines 76–89). In this study, we regarded the presence of pathologically confirmed aldosterone-producing adenoma (APA) as the most important factor. Otherwise, we did not add the ARR values or the results of the confirmatory tests to the inclusion criteria because unsuppressed PRA itself decreases the ARR. However, all eight of the patients who completed the saline loading tests (which does not include PRA in its criterion) in the unsuppressed PRA group showed a positive result (lines 373–375), suggesting hyperaldosteronsim does exist. One patient (patient 3) was exempted from the saline loading test due to the risk of severe hypertension, as described in the response to the previous comment.

We emphasize that the take-home message of this study is the difficulty in diagnosing PA in those with unsuppressed PRA. Therefore, confirmatory tests that include PRA are not suitable inclusion criteria for the unsuppressed PRA group, and we used the presence of pathologically confirmed APA as an important inclusion criterion.

Even if we were to exclude patient 3, who did not have a confirmed positive result in the confirmatory tests, the main result would not differ. Incidentally, patient 3 did fulfill the study’s inclusion criteria, including the presence of pathologically confirmed APA.

There explanations have been added on lines 111–125, 166–171, 366–368, and 375-378.

*3) Again in the methods section authors state that ‘biochemical outcomes are excluded’. As we know from several studies, clinical outcome is important but has several confounding factors such as age, duration of hypertension, renal function and sex. This is why PASO criteria recommends assessing biochemical outcome to confirm ‘biochemical cure’ after 6-12 months using ARR or confirmatory testing. Please further clarify this issue.*

Reply:

In this study, we believed that ARR could not be used as an adequate parameter for assessing post-operative outcomes, as previously described. One important reason for this is that the biochemical outcome assessed in the PASO study requires ARR normalization; however, some patients in this study already exhibited a normal ARR even before surgery due to elevated PRA. Therefore, we would not have been able to assess the normalization of ARR in those patients, and we excluded this assessment of biochemical outcomes in this study.

However, biochemical outcomes can also include assessments of the correction of hypokalemia. In terms of hypokalemia, improvements were observed in the present study. Indeed, patients 1, 2, 6, 7, 8, and 9 (i.e., all of the patients exhibiting pre-existing hypokalemia), avoided the need for oral potassium supplementation after surgery. Therefore, a partial biochemical improvement might be suggested in these patients that is distinct from ARR normalization. We have added this explanation to lines 282–285 and 357–361.

*4) A major concern is the small number of patients with unsuppressed renin levels enrolled in the study. Although the results are in part statistically significant, the sample size is very small. Moreover, patients with unsuppressed PRA showed significantly higher rates of diabetes mellitus. Could this have had an impact on the analysis? The authors may consider cumulating some patients and redoing the analysis.*

Reply:

As described in the Materials and Methods section, we were very strict in our selection of patients in the unsuppressed PRA group, which included those with PRA levels of > 1.0 ng/mL/hr measured in any two or more blood samples due to known fluctuations in PRA (J Clin Endocrinol Metab 2003 Jun;88(6):2489–94.). We believe that this severe criterion ensured the scientific validity of the study, despite leading to the small sample size. Indeed, some patients exhibited inconsistent PRA levels across samples, such as the 0.8, 0.7, and 1.5 ng/dL/hr values measured in one patient (in this case, the patient was excluded due to the lack of reproducibility), as mentioned in the Materials and Methods section. To better explain this, we have added sentences on lines 391–394. Besides, only nine patients fulfilled these criteria of the 969 inpatients with suspected PA who were treated at our hospital between 2007 and 2020. This supports the fact that it is difficult to identify and include patients with unsuppressed PRA in studies of this type.

Regarding the influence of diabetes, diabetes itself could be a risk factor for the acceleration of processes that lead to atherosclerosis and chronic kidney disease. The existence of elevated PRA is known to be associated with atherosclerosis and cardiovascular disease in patients with diabetes (Diabetes Care. 2020 Apr;43(4):843–851.). Additionally, the patients with diabetes show the activation of the renin-angiotensin system and the elevation of PRA via chronic kidney disease (Horm Metab Res. 2013 May;45(5):338–43.). Additional text describing this association has been added on lines 327–332.

*5) Suppression of renin and outcome of PA have gained much attention during the past. Besides aldosterone, the amount of dietary salt intake plays a major role for the suppression of renin levels. This is why I am wondering whether authors also assessed 24-hour urinary sodium excretion to address this problem.*

Reply:

Thank you for your comment. To address this concern, we have added data on the 24-hr sodium urine levels in Table 1 and Table S1. There was no difference in sodium excretion between the two groups, probably due to the fact that patients with suspected PA were on a non-sodium restricted diet (routinely 8 g/day) in our hospital. This is described on lines 103–105 and 335–337.

*6) Please add the BP measurement methods to the methods section and refer to the manuscript of Lenders JWM et al. JCEM 2020. This section should be described more in details.*

Reply:

In our hospital, all blood pressure measurements were conducted by the attending physician to ensure the precise and adequate collection of clinical data (J Clin Endocrinol Metab. 2020 Jun 1;105(6):dgaa159.). In particular, the measurements were conducted according to the guidelines of The Japanese Society of Hypertension (Hypertens Res. 2019 Sep;42(9):1235–1481.). We have added text describing these assessments on lines 105–108.

**Reviewer #2:** 
*1. Why did the authors use “an Endocrine Society Clinical Practice Guideline” for the diagnosis of subclinical Cushing’s syndrome (2010) but not “New diagnostic criteria of adrenal subclinical Cushing’s syndrome: opinion from the Japan Endocrine Society” (2018) ?*

Reply:

We apologize for not updating the citation on the diagnosis of subclinical Cushing’s syndrome. We have revised the citation on line 179.

*2. The author should show the pathological data about the atrophy of the attached normal adrenal cortex after removal of the adrenal tumor in 5 PA patients with unsuppressed PRA who had cortisol levels more than 1.8 μg/dL after overnight dexamethasone suppression test.
3. The authors concluded that PA should not rule out by hypertensive patients with unsuppressed PRA, particularly when they have cortisol elevation. This conclusion is misleading. Each cortisol levels in 9 PA patients with unsuppressed PRA was within normal range. Cortisol elevation might mean autonomous cortisol secretion. However, there were not autonomous cortisol secretion in some PA patients with suppressed PRA.
For example, ACTH levels at 8:00 was 59.2 pg/mL and those at 23:00 was 8.7 pg/mL in Patient 8. These data suggest that ACTH was not suppressed by autonomous cortisol secretion and have diurnal change. How do they explain? They should correct a manuscript.*

Reply:

Thank you for providing the meaningful advice in Comments 2 and 3.

Pathologically, there were no findings suggestive of the presence of apparent atrophy of the attached normal cortex in the unsuppressed PRA group. A previous study (Eur J Endocrinol. 2011 Apr;164(4):447–55.) reported that only 37.1% of aldosterone and cortisol co-secreting adrenal tumors demonstrated evidence of cortical atrophy in the adjacent non-neoplastic tissue. Therefore, patients with aldosterone and cortisol co-secreting adrenal tumors may display unique clinical and endocrinological features. Based on these reports, we have added additional sentences describing the tissue pathology on lines 245–246.

In addition, as Reviewer 2 noted, there were some patients in the unsuppressed PRA group who still maintained diurnal variation. Therefore, the wording of “when they have cortisol elevation” has been revised and alternative phrasing referring to “higher cortisol levels in the 1 mg DST” is now used. Also, the sentences about the patients who did not fulfill the criteria for SCS (Endocr J. 2018 Apr 26;65(4):383-393.) have been modified based on the guideline. The wording in the abstract has also been revised accordingly (line 46–47).

Regarding the diurnal variation, not all of the patients with a positive result in the 1 mg DST (cortisol level ≥1.8 μg/dL) exhibited ACTH suppression and a lack of diurnal variation. Some patients with these endocrinological features have been described even in older SCS guidelines (Endocr J. 2013;60(7):903–12.). A case report about a patient with SCS in whom diurnal variation was maintained has also been published (Endocr J. 2006 Oct;53(5):609–13.). Considering the findings of these reports, those with SCS or patients with a higher cortisol level in the 1 mg DST represent a clinically, pathologically, and endocrinologically heterogenous phenotypes. We have added text describing these findings on lines 296–297, 312, 314–315, 318–325, 326, and 411–412.

*4. There is a possibility that overnight dexamethasone suppression test is false positive in patients with untreated sleep apnea syndrome. BMI in 4 PA patients with unsuppressed PRA was more than 25. Did the authors check sleep apnea syndrome in these patients?
5. The sympathetic nervous activation as well as the decrease in intravascular blood volume increase plasma renin activity. The s**ympathetic nervous activation by untreated sleep apnea syndrome might affect plasma renin activity. The authors should consider this possibility of the mechanism of unsuppressed PRA in addition to autonomous cortisol secretion.*

Reply:

We are grateful for the suggestions and feedback provided in Comments 4 and 5.

We have added data on apnea-hypopnea index (AHI) measurements in Table S1 and additional sentences describing the association between sleep apnea syndrome (SAS) (Sleep. 2009 Dec;32(12):1589–92.), PRA, and cortisol (Clin Endocrinol (Oxf). 2021 Dec;95(6):909–917.) on lines 401–407. In particular, some patients demonstrated a higher AHI, but not all of the patients had undergone screening for SAS, making it difficult to precisely evaluate the relationship (lines 402–408).

*6. The number of PA patients with unsuppressed PRA is few and clinical characteristics of them were also diverse (for example PAC [240 min after saline loading] 14.3-255.0 ng/dL). The authors should discuss the clinical characteristics of each PA patients with unsuppressed PRA.*

Reply:

The diverse clinical characteristics of the patients comprising the unsuppressed PRA group suggest a high degree of heterogeneity. Actually, only six of the patients exhibited higher cortisol levels after the 1 mg DST. Other known PRA-elevating factors are a lower eGFR indicative of chronic kidney disease (eGFR < 60 mL/min/1.73 m^2^), which was observed in three patients, and diabetes, which was present in five patients. Patient 2, in particular, had a prior history of stroke and a long duration of hypertension (21 years).

These multiple factors could have contributed to the elevation of PRA, and further investigations, including a higher number of patients, might elucidate the associations of each factor. Additional details describing the influence of such factors on PRA and PAC have been added to the manuscript (lines 380–389）.

Thank you very much for your thoughtful consideration. We hope we have addressed your concerns satisfactorily. We believe this topic will be of interest to your readers worldwide, and we look forward to your editorial decision.

Please let us know if you have questions or additional suggestions. Thank you.

Yuya Tsurutani

Endocrinology and Diabetes Center, Yokohama Rosai Hospital, Japan 3211 Kozukue-cho, Kouhoku-ku, Yokohama, Kanagawa, 222-0036, Japan.

Tel: +81-45-474-8111

Fax: +81-45-474-8323

e-mail: yuya97tsuru1055@gmail.com
